# Recurrent Septic Shock in Immunosuppressed Patients

**DOI:** 10.1002/ccr3.71249

**Published:** 2025-10-15

**Authors:** Shinnosuke Fukushima, Koji Fujita, Hideharu Hagiya

**Affiliations:** ^1^ Department of Infectious Diseases Okayama University Hospital Okayama Japan; ^2^ Department of General Medicine and Infectious Diseases Tsuyama Chuo Hospital Okayama Japan

**Keywords:** bacteremia, compromised host, cytomegalovirus, septic shock

## Abstract

Cytomegalovirus gastroenteritis presents with diarrhea and abdominal pain in immunosuppressed patients, and histopathological examination is essential by endoscopy. This case illustrates that cytomegalovirus enteritis may develop insidiously and possibly invoke shock in immunocompromised patients, warranting its inclusion in the differential diagnosis of recurrent septic shock.

A 70‐year‐old woman with rheumatoid arthritis presented with fever and was admitted to our hospital. Her medication included prednisolone 4 mg daily and iguratimod, with a notable history of two hospitalizations within the preceding 2 months for septic shock without bacteremia. On admission, the patient exhibited hypotension (78/34 mmHg) and hyperpyrexia (42.0°C), and cefepime was empirically initiated for septic shock. Blood cultures obtained on admission yielded *Escherichia coli* the following day. Contrast‐enhanced computed tomography was performed to evaluate the gastrointestinal tract as a potential source, demonstrating enteritis predominantly involving the terminal ileum (Figure 1A,B). At the time of previous septic shock 1 month ago, colonoscopy revealed ulcerative lesions from the terminal ileum to the ileocecal valve (Figure 1C). The previous colonoscopy findings, showing a terminal ileum ulcer in an immunosuppressed patient, prompted consideration of cytomegalovirus (CMV) infection. Immunostaining was performed on the previous biopsy specimen, and CMV‐positive cells were detected, indicating that CMV enteritis was considered the etiology of recurrent septic shock. CMV antigenemia was negative at the time of admission. Ganciclovir therapy was initiated, and the patient was discharged without further episodes of infection.

**FIGURE 1:**
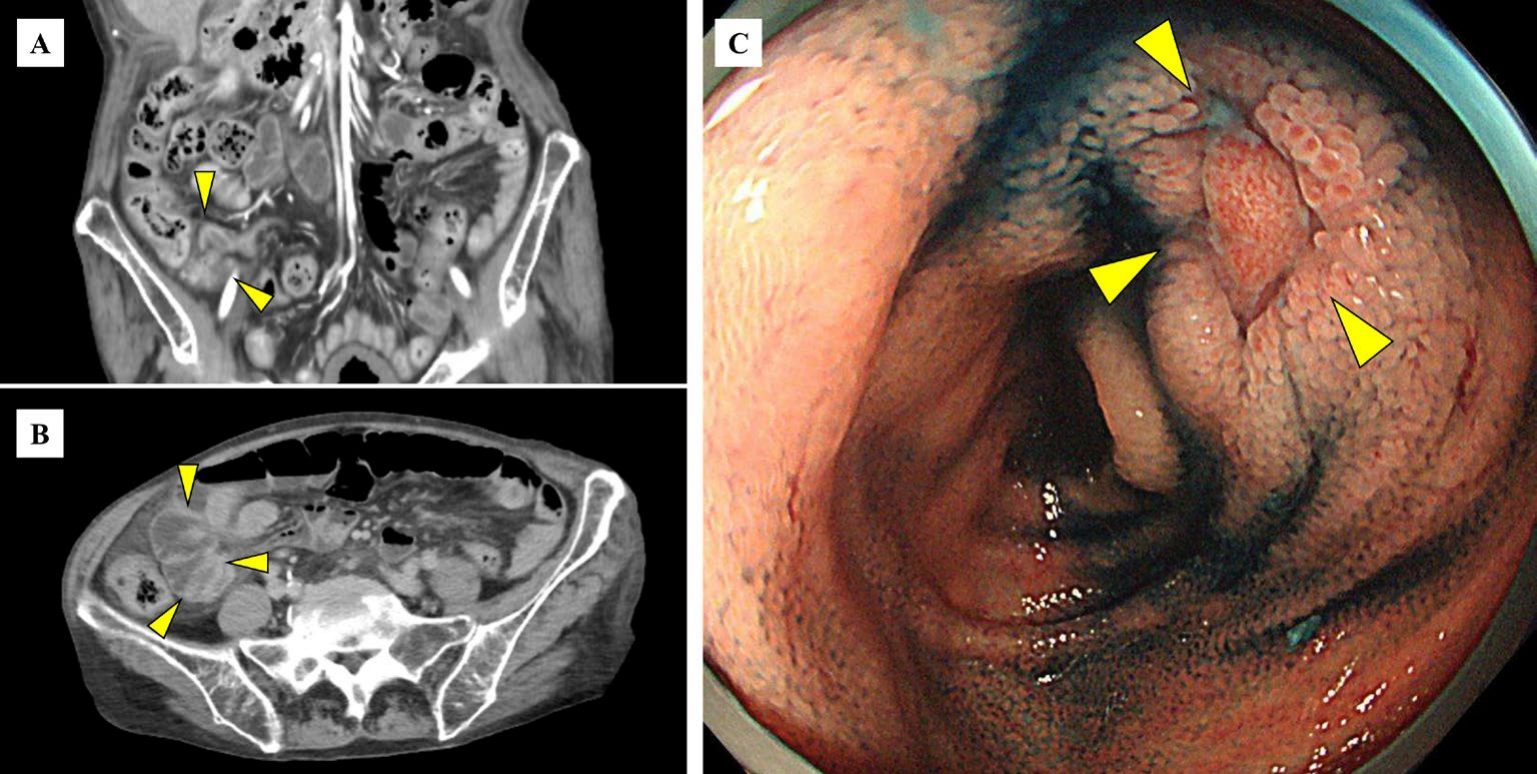
Finding of image. (A, B) Contrast‐enhanced computed tomography (CT). (C) Colonoscopy. (A, B) Abdominal contrast‐enhanced CT shows enteritis predominantly involving the terminal ileum with a contrast enhancing mesentery (yellow arrowheads). (C) Colonoscopy shows an ulcer (yellow arrowheads) located in the terminal ileum.

CMV gastroenteritis presents with gastrointestinal bleeding, diarrhea, and abdominal pain in immunosuppressed patients [1, 2]. Because the accuracy of serodiagnosis is poor for CMV gastrointestinal tract disease, histopathological examination is essential by endoscopy [1, 2]. Intestinal ulcers are observed in 82.5% of immunosuppressed patients with CMV gastroenteritis [2], which has the potential for the development of bacteremia by bacterial translocation. In immunocompetent critically ill patients of the intensive care unit, septic shock had been reported to precede CMV colitis in 92.8% of patients [3]. Our patient presented with recurrent septic shock without gastrointestinal bleeding or diarrhea, which differs from the more common manifestations of CMV enteritis reported previously [1, 2]. Elderly and immunocompromised patients are more frequently colonized by multidrug‐resistant bacteria, making these pathogens an important consideration in the differential diagnosis of recurrent sepsis; however, CMV should also be considered, as in this case. While management guidelines for CMV infection are well established in solid organ transplant recipients, confirmed recommendations for non‐HIV immunocompromised patients receiving immunosuppressive therapy for rheumatologic diseases remain lacking. This case illustrates that CMV enteritis may develop insidiously and possibly invoke shock in immunocompromised patients, warranting its inclusion in the differential diagnosis of recurrent septic shock.

## Author Contributions

**Shinnosuke Fukushima:** conceptualization, writing – original draft. **Koji Fujita:** writing – review and editing. **Hideharu Hagiya:** writing – review and editing.

## Ethics Statement

This case report was reviewed and considered exempt from full Institutional Review Board approval according to our institution's guidelines.

## Consent

Written informed consent was obtained from the patient for the publication.

## Conflicts of Interest

The authors declare no conflicts of interest.

## Data Availability

The datasets used during the current study are available from the corresponding author upon reasonable request.
